# In Vivo Differences in Inputs and Spiking Between Neurons in Lobules VI/VII of Neocerebellum and Lobule X of Archaeocerebellum

**DOI:** 10.1007/s12311-015-0654-z

**Published:** 2015-03-05

**Authors:** Laurens Witter, Chris I. De Zeeuw

**Affiliations:** 1 Netherlands Institute for Neuroscience, Royal Academy for Arts and Sciences (KNAW), Meibergdreef 47, 1105 BA Amsterdam, The Netherlands; 2 Department of Neuroscience, Erasmus Medical Center, Dr. Molewaterplein 50, 3015 GE Rotterdam, The Netherlands

**Keywords:** Cerebellar cortex, Neurophysiology, Patch clamp, Whole cell recording in vivo

## Abstract

The cerebellum plays an important role in the coordination and refinement of movements and cognitive processes. Recently, it has been shown that the main output neuron of the cerebellar cortex, i.e., the Purkinje cell, can show a different firing behavior dependent on its intrinsic electrophysiological properties. Yet, to what extent a different nature of mossy fiber inputs can influence the firing behavior of cerebellar cortical neurons remains to be elucidated. Here, we compared the firing rate and regularity of mossy fibers and neurons in two different regions of cerebellar cortex. One region intimately connected with the cerebral cortex, i.e., lobules VI/VII of the neocerebellum, and another one strongly connected with the vestibular apparatus, i.e., lobule X of the archaeocerebellum. Given their connections, we hypothesized that activity in neurons in lobules VI/VII and lobule X may be expected to be more phasic and tonic, respectively. Using whole-cell and cell-attached recordings in vivo in anesthetized mice, we show that the mossy fiber inputs to these functionally distinct areas of the cerebellum differ in that the irregularity and bursty character of their firing is significantly greater in lobules VI/VII than in lobule X. Importantly, this difference in mossy fiber regularity is propagated through the granule cells at the input stage to the Purkinje cells and molecular layer interneurons, ultimately resulting in different regularity of simple spikes. These data show that the firing behavior of cerebellar cortical neurons does not only reflect particular intrinsic properties but also an interesting interplay with the innate activity at the input stage.

## Introduction

The cerebellum directs motor learning and might be involved in a myriad of other processes, including emotion and cognition [1–3]. These differential behavioral roles are expected to be reflected in the specific characteristics of the network function of the particular cerebellar cortical region involved, in that processing in its neurons may be tailored to the specific task at hand. Indeed, Purkinje cells, the sole output neurons of the cerebellar cortex, show different intrinsic properties and different activity levels in several distinct areas of the cerebellar cortex, often related to a specific zonal distribution [4, 5]. For example, Purkinje cells in the zebrin-positive zones of the vestibulocerebellum show a lower firing rate and have a tendency to be more regular than those in the largely zebrin-negative zones of the anterior cerebellum [4]. Similarly, mossy fiber inputs to the various cerebellar cortical regions serving functions with inherently different dynamics can also be expected to show differences in activity patterns [6–9]. However, to what extent differences in regularity and firing rates of mossy fiber inputs penetrate into the cerebellar network from the input stage to the output stage remains to be explored in detail. Here, we sought to investigate differences in the activity of mossy fibers, granule cells, Purkinje cells, and interneurons between two functionally distinct areas of the cerebellar cortex, lobules VI/VII of the neocerebellum and lobule X of the archaeocerebellum. Lobules VI/VII are intimately connected to sensorimotor and frontal cortices controlling active exploratory behavior with the use of fast whisker, paw, and/or eye movements [10–14]. Lobule X forms an integral part of the vestibulocerebellum receiving inputs mainly from the vestibular organs and controlling slower reflexive trunk movements. Both frequency and regularity of mossy fiber inputs, as recorded in granule cells, were significantly different between lobules VI/VII and lobule X. When looking at output of molecular layer interneurons, the activity pattern of which is dominated by the parallel fiber inputs from the granule cells, we observed that in accordance with mossy fiber inputs, spiking was more regular in lobule X compared with lobules VI/VII. Similarly, simple spike (SS) firing in Purkinje cells, the intrinsic activity which is modulated by both granule cell and molecular layer interneuron inputs [15, 16], was more regular in lobule X compared with lobules VI/VII. Together, our data show that there can be considerable differences in mossy fiber activities between lobules and subsequent spiking of granule cells, Purkinje cells, and molecular layer interneurons. These differences can be lobule-specific and probably reflect the specific needs of the cerebellar cortex in processing different modalities.

## Materials and Methods

Adult (4–8 weeks) C57Bl/6 mice were prepared for whole cell recordings in vivo by placing a pedestal on the skull during a preparatory surgery. In short, animals were anesthetized using isoflurane (5 % induction, 1.5 % in 0.5 l/min O_2_ and 0.2 l/min air), while body temperature was kept constant at 37 °C via a feedback-controlled heating pad. The skin was shaved and incised for approximately 1 cm midsagitally on the skull. The bone was etched (37.5 % phosphoric acid, Kerr), and a primer (Optibond, Kerr) was applied before a pedestal containing two M1.4 nuts was glued to the skull using dental acrylic (Flowline, Hereaus Kulzer). Animals received pain reliever in the form of 2 mg/kg metacam (AUV). Animals were allowed to recover for at least 1 day before recordings were performed. All experiments adhered to institutional and national guidelines and were approved by the Dutch Animal Ethical Committee.

### Electrophysiology

On the day of the experiment, the mouse was anesthetized with an i.p. injection of 75 and 12 mg/kg ketamine and xylazine, respectively. Anesthesia was supplemented when needed during the experiment. The occipital bone was exposed by removing the skin and three muscle layers in the neck of the mouse. For the stability of the recordings, we usually made small holes in the bone so as to reach lobules VI/VII or lobule X. When necessary, we used 4 % agar to stabilize the electrode to the brain. Whole-cell recordings of all neurons were made using filamented borosilicate glass (Hilgenberg or Harvard apparatus, 1.5 mm OD, 0.86 ID), and pipettes were filled with (in mM): 10 KOH, 3.48 MgCl2, 4 NaCl, 129 K-Gluconate, 10 HEPES, 17.5 glucose 4 Na2ATP, and 0.4 Na3GTP (295–305 mOsm; pH 7.2). For recordings in lobules VI/VII, the electrode was lowered down to 200 μm with high pressure on the electrode, before agar was applied to stabilize the recording electrode to the brain. For recordings in lobule X, the electrode was quickly lowered to depths between 1,500 μm before the pressure was lowered. “Neuron hunting” was done by making 2-μm steps under low (15–30 mbar) pressure. The total distance the electrode traveled from the surface of the brain was noted for all recordings (track length). Recordings were amplified using a Multiclamp 700B amplifier (Axon Instruments) and digitized between 10 and 50 Khz using a Digidata 1440 (Axon Instruments). Bridge balance and capacitance neutralization was employed for all recordings. Junction potential between the electrode and extracellular milieu was determined to be −8.53 ± 0.87 mV. In whole cell mode, a 10 mV pulse was applied and the resulting current response was recorded. Access resistance and membrane resistance were estimated from the peak and steady-state responses, respectively, whereas membrane time constants were estimated from the relaxation from peak to steady state. Analysis was performed using Clampfit (Axon Instruments, version 10.2), Matlab 2010b (The Mathworks), and Excel. Reported numbers in the text are mean ± SD, unless noted otherwise. At the beginning of each section, the total number of analyzed neurons is listed. This neuron number applies to all analyses in that section unless indicated otherwise.

### EPSP Detection

Excitatory postsynaptic potentials (EPSPs) were detected by obtaining the finite difference with an interval of 20 μs to 1 ms [approximating a differential with variable Δ*t* in the form f(*t*_2_) − f(*t*_1_), where Δ*t* = *t*_2_ − *t*_1_]. A threshold was then set to detect the rising phase of the EPSPs, and EPSP peaks were detected in the following 2 ms. The baseline was determined just before onset of the EPSP. Visual inspection was carried out on all traces and events. Due to the much higher noise levels of in vivo recordings compared with in vitro recordings, some events could not be reliably classified and were not included. On average, we could detect 85–90 % of all events, without including nonevents.

### SAR Analysis

Simple spikes, in contrast to complex spikes (CSs), are generated by persistent and resurgent sodium currents at the axon initial segment [17, 18]. Since the complex spike is generated in the dendritic tree [19], it has a much larger dendritic component than simple spikes. We used this difference to estimate whether our recordings were made from the perisomatic or dendritic region of the Purkinje cell involved. Dividing simple spike amplitude by complex spike amplitude [spike amplitude ratio or SAR] was a good indication of recording location, since there was a clear relation between the SAR and recording depth in lobules VI/VII (*R* ^2^ = 0.52). Half-widths of simple spikes were calculated when SAR > 0.8 to avoid dendritically filtered simple spikes.

### Histology

A subset of neurons was filled with 0.5 % Neurobiotin (Vector labs). Since Neurobiotin has previously been reported to alter membrane currents in some neurons [20], we tested a subset of cells without injections of Neurobiotin. In none of the cell populations did we detect a difference between recordings with and without Neurobiotin. After successful retraction of the electrode from the cell, as indicated by obtaining an outside-out patch, the animal was perfused with 0.5 % paraformaldehyde and 2.5 % glutaraldehyde in 0.11 M phosphate buffer containing 4 % sucrose. The brain was then postfixed for 1 night at 4 °C in the same solution, after which it was cut into 100-μm-thin slices and stained either with avidin–biotin complex (Vector labs) followed by DAB staining or with streptavidin–Alexa conjugate (Life Technologies). Neurons labeled with ABC-DAB and streptavidin–Alexa conjugate were visualized using a normal transmission-light microscope or a confocal microscope (Leica, SP5), respectively.

## Results

**Fig. 1:**
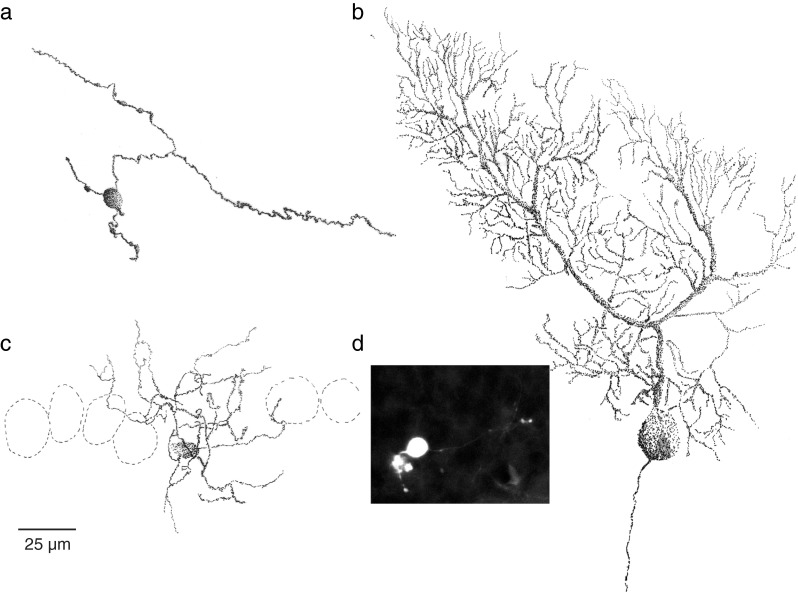
Reconstructions of recorded neurons. **a** Granule cell. The soma is approximately 8 μm across. The ascending branch of the parallel fiber can be observed leaving the soma at the 1 o’clock position. Two dendrites can be observed at the 6 and 10 o’clock positions. **b** Purkinje cell. The typical large soma and dendritic tree were reconstructed. **c** A basket cell was reconstructed. The axon and dendrites could not be readily distinguished and are both shown in *black*. Outlines indicate the Purkinje cell layer. **d** Confocal z-stack projection of a recovered unipolar brush cell. The typical brush-like dendrite can be seen at the *left* of the cell body, while the axon can be seen leaving the cell at the *right*

**Fig. 2:**
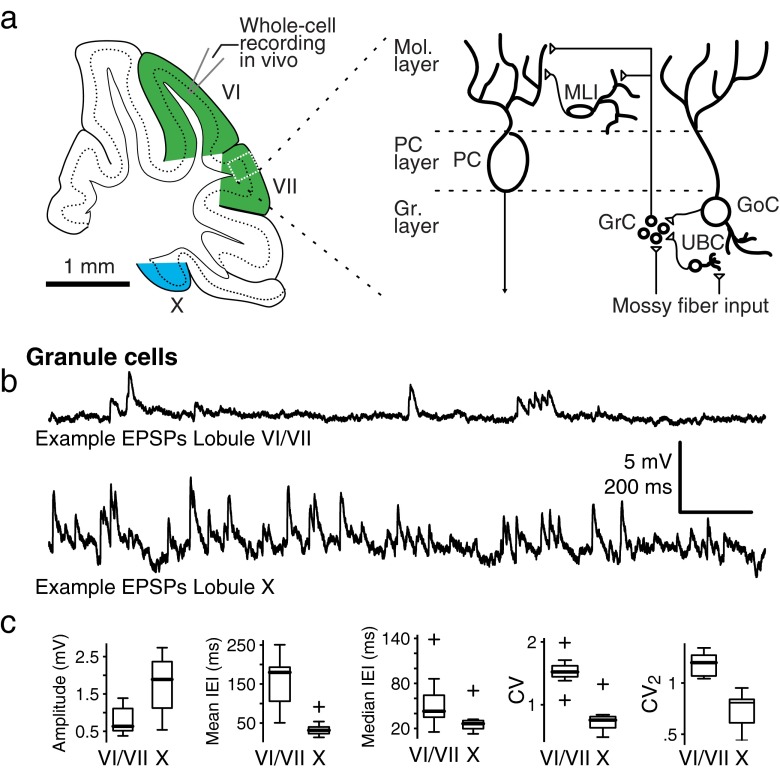
Granule cells. **a** *Left*: Schematic overview of the experiment. Cells were patched in either lobules VI/VII (*green*) or lobule X (*blue*) (Adapted from Ref. [50]). *Right*: Schematic overview of the cerebellar circuitry, indicated are the Purkinje cell (*PC*), molecular layer interneuron (*MLI*), granule cell (*GrC*), Golgi cell (*GoC*), and unipolar brush cell (*UBC*). Dendrites are indicated with *thick lines*, axons with *thin lines*. Synapses are indicated with a *triangle*. **b** Two examples of granule cell subthreshold activity in lobules VI/VII (*left*) and lobule X (*right*). **c** Boxplots of EPSP propertiesGranule cells. **a** *Left*: Schematic overview of the experiment. Cells were patched in either lobules VI/VII (*green*) or lobule X (*blue*) (Adapted from Ref. [50]). *Right*: Schematic overview of the cerebellar circuitry, indicated are the Purkinje cell (*PC*), molecular layer interneuron (*MLI*), granule cell (*GrC*), Golgi cell (*GoC*), and unipolar brush cell (*UBC*). Dendrites are indicated with *thick lines*, axons with *thin lines*. Synapses are indicated with a *triangle*. **b** Two examples of granule cell subthreshold activity in lobules VI/VII (*left*) and lobule X (*right*). **c** Boxplots of EPSP properties

Whole cell in vivo recordings were made from neurons in lobules VI/VII and lobule X of the cerebellar cortex (Figs. 1 and 2a). There was no difference in the success rate of obtaining a patch between these areas. Each cerebellar cortical cell type showed characteristic suprathreshold and subthreshold activities and characteristic responses to current input. We used such activities and input–output responses to identify every neuron type and verified this with morphological identification [21, 22].

### Granule Cells

To identify differences in mossy fiber inputs between lobules VI/VII and lobule X, we patched 13 granule cells in lobules VI/VII and 10 in lobule X of the cerebellar cortex in intact anesthetized mice (Figs. 1a and 2b; Table 1). All granule cells patched were initially silent during the recording; the cells only started to fire action potentials when the quality of the recording deteriorated (Fig. 2b). Moreover, in cell-attached mode, before breaking into the cell, we never observed spontaneous action potentials. Therefore, output from granule cells was not analyzed from granule cell recordings, but only indirectly from inputs to molecular layer interneurons (see below). As can be expected from small cells, the granule cells showed a high input resistance and short membrane time constants (Table 1). We found small, insignificant, differences in membrane parameters between granule cells in lobules VI/VII and lobule X (Table 1). Subthreshold activity in granule cells was dominated by fast EPSPs arriving at an average frequency of 20.8 ± 4.7 (Fig. 2b, c). Granule cells in lobules VI/VII showed significantly less of these events than those in lobule X (lobule VI/VII vs. lobule X 6.9 ± 1.8 Hz vs. 26.9 ± 6.7 Hz, *p =* 0.01; Fig. 2b, c). Moreover, in cells of lobules VI/VII, the distribution of interevent intervals (IEI; i.e., in between mossy fiber events) was significantly more irregular than in cells in lobule X (CV lobule VI/VII vs. lobule X 1.53 ± 0.07 vs. 0.77 ± 0.08, *p* ≪ 0.001; Fig. 2b, c). When depolarized, all granule cells fired action potentials (average width at half-amplitude 0.88 ± 0.49 ms). Due to the high resistance of the cells, small currents were sufficient to drive vigorous spiking in cells. Interspike intervals shorter than 10 ms were frequently observed and could be as short as 2.1 ms. Shortest intervals were not different between lobules VI/VII and X (mean shortest intervals lobule VI/VII vs. lobule X: 5.5 ± 3.2 ms vs. 8.1 ± 4.9 ms, *p* = 0.34). Granule cells fired spikes tonically with a linear current input–firing frequency relation (*R* ^2^ > 0.74 for all cells, mean *R* ^2^ = 0.93), and there was no difference in linearity of the responses between lobule VI/VII and lobule X (*R* ^2^: lobule VI/VII vs. lobule X 0.96 ± 0.06 vs. 0.97 ± 0.03, *p* = 0.80). Similarly, there was no difference in the slope of the current input-firing frequency relation (slope lobule VI/VII vs. lobule X 4.3 ± 1.5 Hz/pA vs. 4.3 ± 2.0 Hz/pA, *p* = 0.98).

**Table 1:** Cell physiological parameters of all recorded neurons

		Rm (MΩ)	Tau (ms)	FF (Hz)	N
Granule cells	VI/VII	778 ± 80	0.38 ± 0.02	0	13
	X	583 ± 53	0.35 ± 0.03	0	10
Purkinje cells	VI/VII	41 ± 10	7.39 ± 1.09	66.4 ± 6.8	13
	X	108 ± 75	31.15 ± 22.68	45.3 ± 12.1	6
MLIs	VI/VII	139 ± 30	2.12 ± 0.27	9.8 ± 3.6	12
	X	147 ± 19	4.75 ± 0.78	5.5 ± 1.9	20
Golgi cells		459 & 578	2.52 & 3.37	24.9 & 14.6	2
UBCs		400 ± 68	1.85 ± 0.70	7.3 ± 3.5	4

### Purkinje Cells

**Fig. 3:**
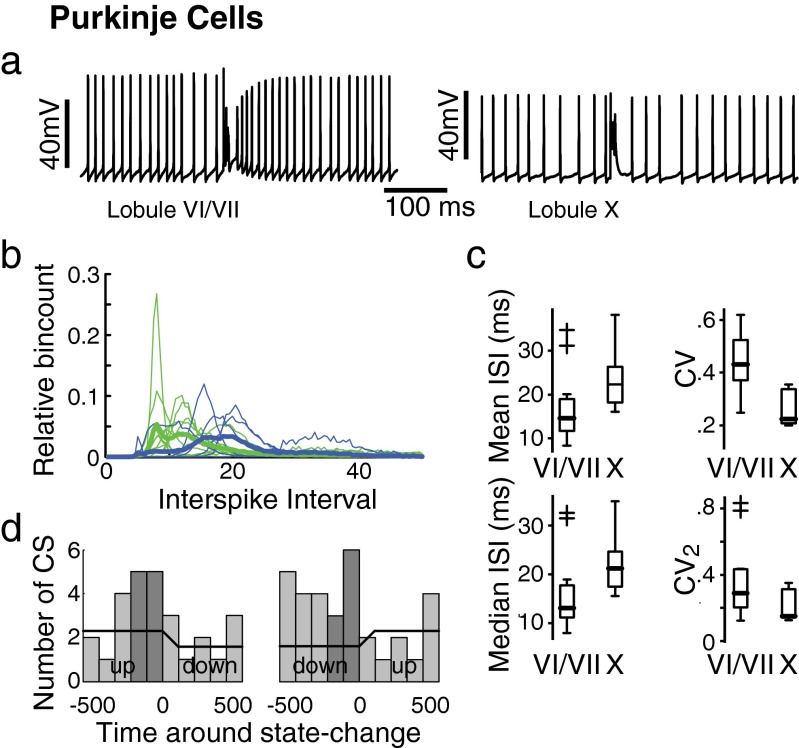
Purkinje cells. **a** Two examples of simple and complex spike (*center*) firing in lobules VI/VII (*left*) and lobule X (*right*). **b** Histograms of interspike intervals of simple spikes for all cells (*thin lines*) for both lobules VI/VII (*green*) and lobule X (*blue*). *Thick lines* indicate the average interspike interval distributions. **c** Boxplots of spiking activity of simple spikes. **d** Histograms of complex spikes around state changes. Upstate to downstate switches (*left*) and downstate to upstate switches (*right*) are indicated. Dark grey bars indicate the time window [−200 ms:0 ms]

To evaluate the final output stage of cerebellar cortical processing, we recorded from Purkinje cells in lobules VI/VII (*N* = 13) and lobule X (*N* = 6) (Fig. 1b). Purkinje cells are the principal neuron of the cerebellar cortex and provide its sole output. They could be identified by their low membrane resistance, high capacitance (Table 1), and characteristic simple spike (SS) and complex spike (CS) firing in vivo (Fig. 3a). Simple spikes, which are generated by a persistent and resurgent sodium currents at the axon initial segment [17, 18], showed the typical fast single-spike waveform with a short width at half-amplitude (half-width 0.33 ± 0.07 ms for cells with SAR > 0.8). In contrast, complex spikes, which are generated by climbing fiber input impinging on the proximal dendrites of the Purkinje cells [19], showed the typical multiple spikelets riding on top of a pronounced calcium plateau (Fig. 3a). In addition, Purkinje cells in both lobules VI/VII and lobule X showed the typical switch between a depolarized and hyperpolarized state (i.e., bistability) that often occurs under anesthesia [23, 24]. The average membrane potentials during upstate and downstate were −42.8 ± 3.9 and −55.8 ± 7.3 mV (*t* test *p* < 0.001), respectively. During the upstate, all recorded Purkinje cells fired both simple spike and complex spikes (SS 62.5 ± 23.4 Hz, range 27.6–121.0 Hz; CS 0.70 ± 0.32 Hz, range 0.2–1.3 Hz), whereas during downstates, only complex spike activity was observed. Spontaneous bistability could be observed and quantified in nine cells, but in all of the remaining cells (*N* = 10), downstates could be induced by hyperpolarizing currents. In both lobules VI/VII and lobule X, complex spikes could induce state transitions, but state transitions could also occur without an associated complex spike. As expected from literature [23, 25], switches from upstate to downstate were generally slow and were preceded by a complex spike within 200 ms in 29 % of the cases (10 out of 34 state changes; Fig. 3d). Downstate to upstate switches were preceded within 200 ms by a complex spike in 24 % of the state changes (9 out of 37 state changes; Fig. 3d). Despite the relatively high change of complex spike activity before a state change, the chance of a complex spike inducing a state change was lower than 1 % for both upstate-to-downstate and downstate-to-upstate changes (10 and 9 CSs, respectively, associated with a state change compared with 1,329 CSs in total). Because of the relatively low number of state changes and the low number of cells analyzed, we did not compare state changes between lobules VI/VII and lobule X. Combining upstate and downstate, complex spike firing rates were relatively low and not significantly different (*p* = 0.82) between lobules VI/VII (0.67 ± 0.31 Hz) and lobule X (0.72 ± 0.37 Hz). In contrast, simple spike activity showed differences among the lobules. In accordance with the differences in granule cell activity described above, simple spike firing during upstates in lobules VI/VII was more irregular than in lobule X (0.44 ± 0.14 vs. 0.25 ± 0.09, respectively, *p* = 0.02; Fig. 3c). Firing rates during upstates did not differ between lobules, but there was a trend for slightly lower firing rates in lobule X (lobules VI/VII 66.4 ± 24.5 Hz vs. lobule X 47.1 ± 12.6 Hz, *p* = 0.12; Fig. 3b, c).

### Molecular Layer Interneurons

**Fig. 4:**
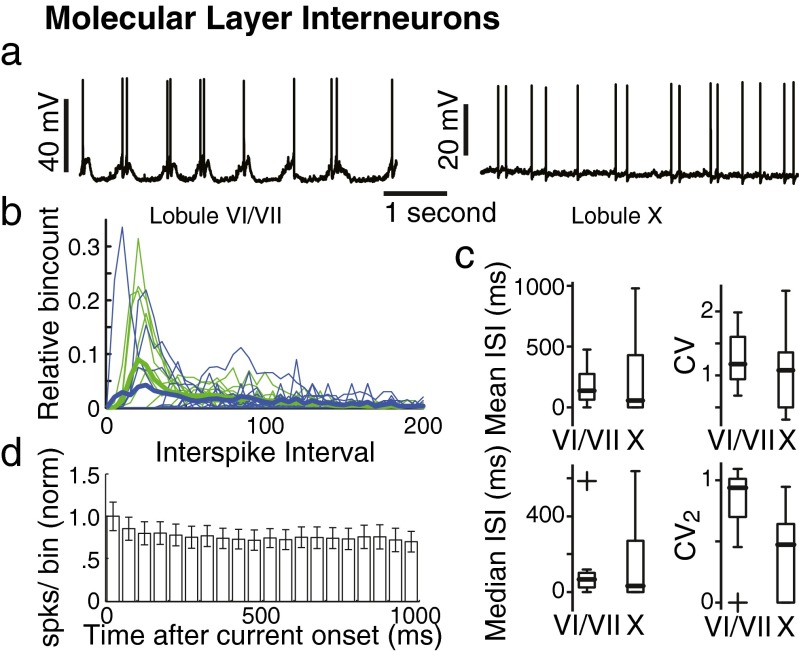
Molecular layer interneurons. **a** Two examples of spiking activity of molecular layer interneurons in lobules VI/VII (*left*) and lobule X (*right*). **b** Histograms of interspike intervals for all cells (*thin lines*) for both lobules VI/VII (*green*) and lobule X (*blue*). Thick lines indicate the average interspike interval distributions. **c** Boxplots of spiking activity of molecular layer interneurons. **d** Firing rate adaptation over a 1,000 ms current input. Each bin represents the normalized (to bin 1) number of spikes fired in 50 ms. Error bars indicate ± SEM

Purkinje cell activity is regulated not only by granule cell input but also by molecular layer interneurons, which provide a feed-forward inhibition from granule cells onto Purkinje cells [16, 26]. Additionally, molecular layer interneurons receive spill-over climbing fiber input and sense extracellular calcium to provide feed-forward inhibition in response to climbing fiber input and synaptic activity, in general [27–29]. To further evaluate the output of granule cells and its possible impact on Purkinje cells, we recorded from molecular layer interneurons, which are electrically more compact than Purkinje cells and thus provide an opportunity to record granule cell output in the form of EPSPs. We recorded the activity of 32 molecular layer interneurons from lobules VI/VII (*N* = 12) and lobule X (*N* = 20) in anesthetized mice. Molecular layer interneurons were characterized by low membrane resistance (144.3 ± 90.6 MΩ) and intermediate membrane time constants (3.8 ± 3.1 ms; Table 1). Irrespective of their location in lobules VI/VII or lobule X, the molecular layer interneurons all received spontaneous excitatory synaptic inputs. Due to the low amplitude and high frequency of synaptic inputs, it was impossible to reliably identify separate events (Fig. 4a). Given that most of the granule cells were silent in our preparation (see above), these findings imply that molecular layer interneurons probably receive input from a large population of granule cells [9, 30]. Although we could not reliably analyze individual EPSPs, we observed that molecular layer interneurons recorded from lobules VI/VII received excitatory inputs arriving in bursts, whereas those in lobule X occurred in a tonic fashion (Fig. 4a). Indeed, when comparing the regularity of molecular layer interneuron spiking to mossy fiber inputs to granule cells, we observed that spiking in lobules VI/VII interneurons was more irregular than that in lobule X; this held especially true at the shorter time scales (CV 1.15 ± 0.33 vs. 0.98 ± 0.54, *p* = 0.36; CV_2_ 0.93 ± 0.15 vs. 0.64 ± 0.19, *p* = 0.001; Fig. 4b). Together, these data indicate that the level of regularity of mossy fiber inputs to granule cells is probably largely conserved in the output of the granule cells.

Since molecular layer interneurons inhibit Purkinje cells, shaping their activity, we also recorded spike output from molecular layer interneurons. Spontaneous action potential firing in molecular layer interneurons was highly irregular (CV 1.1 ± 0.5) and varied greatly between cells (firing frequency 7.1 ± 10.2 Hz, range 0–43.8 Hz), but was not different between recording locations (lobule VI/VII vs. lobule X CV: 1.15 ± 0.33 vs. 0.98 ± 0.54; *p =* 0.36; Fig. 4). Also in cell-attached configuration, there was a high spread of activity profiles (firing frequency 4.1 ± 6.6 Hz, range 0–23.4 Hz, *N* = 14) and a high degree of irregularity in the spike trains (CV 1.4 ± 0.5), but there was no significant difference between whole cell and cell-attached modes in either firing frequency (*p =* 0.213) or regularity (*p =* 0.22). Moreover, with regard to the location, we also did not observe any difference between lobules VI/VII and lobule X with respect to the firing frequency (9.8 ± 12.6 Hz vs. 5.5 ± 8.4 Hz; *p* = 0.25). Like in granule cells, the relation between one second input current and firing frequency was linear in molecular layer interneurons and not different between lobules VI/VII and X (*r* = 0.94 ± 0.12; slope 0.49 ± 0.51 Hz/pA, range 0.34–2.39 Hz/pA; *p* = 0.15). After the first 50 ms of 1 s current input, there was a significant adaptation in firing frequency [ANOVA *F*(19,570) = 4.23, *p* < 0.001; Fig. 4d]. This indicates that although molecular layer interneurons respond linearly to inputs on a shorter timescale, for longer activations, the firing frequency is attenuated. Again, there was no difference between lobules VI/VII and lobule X in this respect (*p* = 0.23), highlighting that the main characteristics of the spatiotemporal patterns of the simple spike activity are relayed from the mossy fiber inputs to granule cells and via their parallel fibers directly onto the Purkinje cells, rather than via the molecular layer interneurons.

**Fig. 5:**
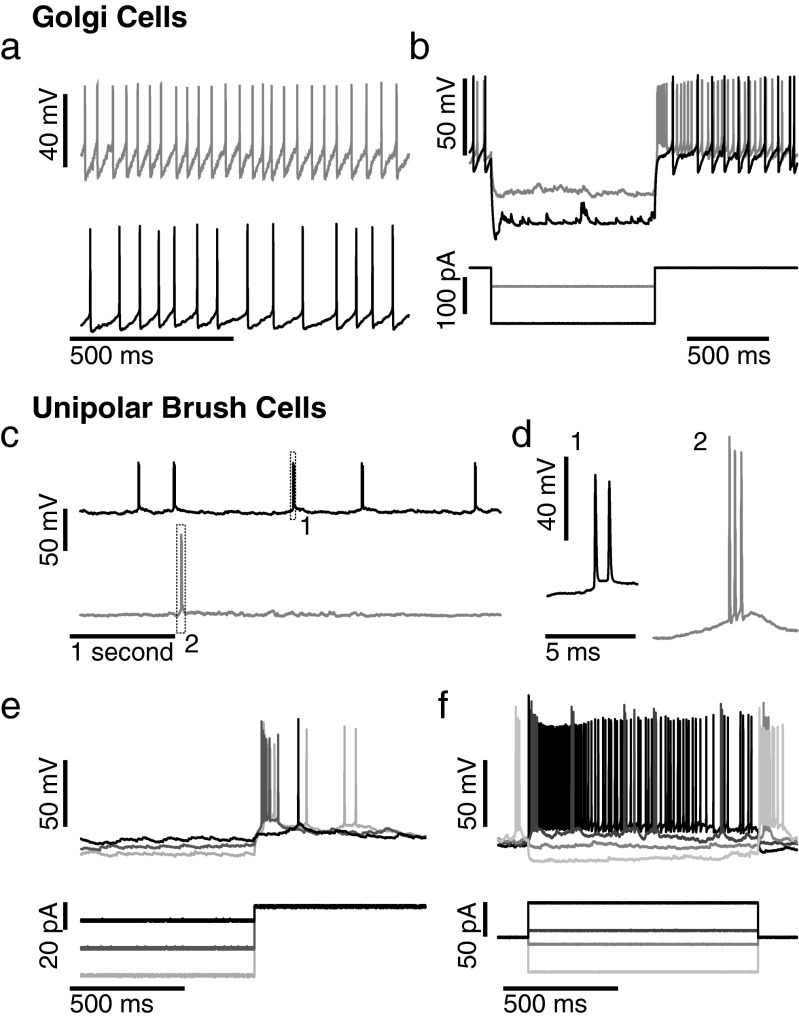
Golgi and unipolar brush cells. **a** Two examples of Golgi cell spontaneous firing. **b** Current inputs in the two cells shown in **a** (same greyscale as in **a**). **c** Two unipolar brush cells show spontaneous burst firing. Two such bursts, as indicated by the numbered boxes, are enlarged in **d. d** Enlargement of the bursts shown in **c. e** Release from hyperpolarization induced burst firing in unipolar brush cells. **f** Depolarization induces tonic spike firing in unipolar brush cells

Membrane properties and spiking activity of molecular layer neurons was clearly different from interneurons recorded in the granule cell layer. Golgi cells (*N* = 2; Table 1), the main interneuron in the granular layer, showed typical regular, spontaneous firing at 10–30 Hz (CV 0.1–0.3), a higher membrane resistance of about 400–600 MΩ, and strong delayed hyperpolarization-activated depolarizing currents and rebound spiking (Fig. 5a, b; Table 1; for comparison with in vitro data see also Refs. [31–36]). Moreover, the activity of the molecular layer interneurons also diverged from that of unipolar brush cells recorded in the granular layers of lobules VI/VII (*N* = 2) and lobule X (*N* = 2), which was characterized by an intermediate membrane resistance (400.4 ± 135.6 MΩ), a short membrane time constant (1.85 ± 0.70 ms), and combined bursty and tonic firing, the latter at a relatively low average firing frequency (7.3 ± 7.1 Hz, ranging from 0 to 16.1 Hz in whole cell mode; 0 and 0.7 Hz for two neurons recorded in cell-attached mode; Fig. 5c, d; Table 1). When these neurons were released from hyperpolarization, a burst was often observed, while at slight depolarized potentials, tonic action potential firing could be observed (Fig. 5e, f; see also Refs. [37–39]).

## Discussion

The target of the here-described research was to establish whether two functionally distinct areas of the cerebellar cortex differ in activity and cell physiological parameters. We found that input from mossy fibers was radically different between lobules VI/VII and lobule X. Input to lobules VI/VII was characterized by bursts of EPSPs to granule cells, while input to lobule X was tonic. This difference was propagated to granule cell spiking as observed by EPSPs recorded in molecular layer interneurons. Ultimately, granule cells and molecular layer interneurons impinge on Purkinje cells to regulate their simple spike firing. As expected, Purkinje cell simple spike firing showed differences in regularity between lobules VI/VII and lobule X, with more irregular spiking in lobules VI/VII compared with lobule X.

Recent research has shown that there are differences in intrinsic membrane properties of Purkinje cells in lobules III–V vs. lobule X, which are part of the paleocerebellum and archeaocerebellum, respectively [5]. We did not observe such differences in cell membrane properties between neocerebellum and archeaocerebellum, highlighting possible specification of the paleocerebellum. Another study investigating differences in membrane properties of cerebellar neurons in paleocerebellum and archeaocerebellum found that the Cav3–Kv4 complex was differentially regulated between lobules II and IX [40]. This difference may have important implications for the manner granule cells interpret their mossy fiber inputs, with granule cells in lobule IX being able to follow slowly changing mossy fiber input rates and granule cells in lobule II being more sensitive to burst inputs. Similar mechanisms may be at play in lobules VI/VII, where granule cells receive inputs in bursts.

It thus seems that there is an intricate interplay between cell physiological parameters and cell input. Likewise, previous work seems to suggest that there are considerable differences in the activities of mossy fibers between cerebellar regions. In the cerebellar flocculus (i.e., hemispheric extension of lobule X) of mice anesthetized with ketamine–xylazine, mossy fibers fire mainly tonically and modulate their firing rate gradually according to vestibular stimuli [9]. In contrast, in the anterior lobe of the cat, mossy fibers seem to respond with bursts of activity upon touch stimuli [6–8]. Here, we report for the first time whole cell recordings from Golgi and unipolar brush cells. Other researchers have recorded parallel fiber input in molecular layer interneurons in whole cell mode previously, but only from neocerebellum and paleocerebellum [29, 41]. In these recordings, irregular but high-frequency parallel fiber inputs were reported, in line with the current findings.

Zebrin striping has been shown to be an important determinant of Purkinje cell activity, with Purkinje cells in zebrin-positive zones showing considerably lower firing rates than those in zebrin-negative zones [4]. The vestibulocerebellum is zebrin-positive, and indeed in our current experiments, we observed a trend toward lower firing rates in Purkinje cells in lobule X compared with those in lobules VI and VII. Interestingly, lobules VI and VII are also mostly zebrin–positive, and this might in part explain the relative lack of difference in firing rates between Purkinje cells in lobules VI/VII and X. Future studies should look into the possibility of an interplay between zebrin striping and mossy fiber afferent regularity to evaluate the relative contributions of Purkinje cell intrinsic activities vs. afferent influences to the irregularity of Purkinje cell firing.

Lobule X of the cerebellum, which is connected to the vestibular apparatus, is involved in balance and related eye movements, whereas lobules VI and VII, which are intimately connected to the cerebral cortex, are involved in paw movements and possibly cognitive processes [42, 43]. The difference in regularity of inputs and outputs of the cerebellar cortex could be explained by an always-on mechanism controlling balance and posture in the vestibular cerebellar vs. a cortical oscillation-based input for lobules VI/VII. Interestingly, previous studies have indicated a close relation between neocortical oscillations and activity in the cerebellum during anesthesia [44, 45], which in part might explain the lower EPSP rates observed in granule cells in lobules VI and VII compared with granule cells in lobule X. Still, it is not expected that all effects observed were due to anesthesia. Previous work has shown differences in the firing rate and regularity of Purkinje cells in unanesthetized mice, with lower Purkinje cell simple spike firing frequencies in lobule X compared with lobules I–III [4]. Moreover, in decerebrate cats, in which pontine inputs are not operating in a functional manner, mossy fiber inputs to granule cells in the C3 zone controlling front paw movements also appear bursty against a low tonic background [8].

### Cell Numbers in the Cerebellar Cortex and Technical Considerations

Apart from investigating differences in activity patterns between two functionally distinct areas of the cerebellum, we also systematically tried to record from all neuronal types in the cerebellum. We patched all major cell types in the cerebellar cortex, Purkinje cells, granule cells, molecular layer interneurons, putative Golgi cells, and unipolar brush cells and characterized their activity. All cell classes showed differences to one another, and we tried to identify each cell type based on their activity profile. We propose a simple scheme to readily identify each cell during in vivo recordings: Purkinje cells are identified by their simple and complex spikes; granule cells are identified by their high input resistance (>350 MΩ) and low capacitance (<10 pF); molecular layer interneurons, in turn, show a lower membrane resistance (<500 MΩ) and higher capacitance (>10 pF); Golgi cells show a higher input resistance (>450 MΩ) and a high capacitance (>25 pF) compared with molecular layer interneurons, and in addition, they show a hyperpolarization-activated depolarizing current with no clear EPSPs; and unipolar brush cells, which have an input resistance of >250 MΩ and capacitance of >15 pF, also show a hyperpolarization-activated depolarizing current, but in addition, a typical burst discharge of action potentials.

There are several factors governing the cell types patched during a given in vivo whole cell experiment. First, the size of the electrode tip is of importance: small tips (~1 μm) select for small cells. Second, the depth at which neuron hunting is started will affect the neurons patched. In general, one can expect to have encountered a cell within 150 μm from the start of the search. Third, the density of the different neuronal classes differs greatly. Therefore, to get an estimate of expected probability of encountering a particular cell type, we calculated for each cell type the estimated total surface membrane in a cubic millimeter of cerebellum (Table 2). From this table, it can be concluded that the chance of encountering a Golgi cell is very low indeed. The chance of encountering a unipolar brush cell in lobule X is more than double the chance of encountering a Golgi cell. Still, in our dataset, there was an overrepresentation of molecular layer interneurons and a underrepresentation of Purkinje cells [*χ* ^2^(4,*N* = 89) = 217.74, *p* ≪ 0.001]. This is probably because we mostly used smaller tipped electrodes, thereby targeting smaller cells like interneurons and unipolar brush cells. Also, starting neuron hunting 200 μm below the pial surface (see “Materials and Methods”) biased the sampled population toward molecular layer interneurons.

**Table 2:** Estimations of neuron densities in the cerebellar cortex

	Density (/mm^3^)	Diameter (μm)	Total surface per cell (μm^2^)	Total surface overall (μm^2^)	Chance overall	Fraction patched
Granule	2.63E + 6^a,b^	5.4^c,d^	9.16E + 1	2.41E + 8	35.0 %	23/69 (33.3 %)
Purkinje	2.00E + 4^e^	80^c^	2.01E + 4	4.02E + 8	58.3 %	18/69 (26.1 %)
MLI	1.00E + 5^b^	10^c,d,f^	3.14E + 2	3.14E + 7	4.6 %	22/69 (31.9 %)
Golgi	4.50E + 3^g,h^	18^c,d,g,h^	1.02E + 3	4.59E + 6	0.7 %	2/69 (2.9 %)
UBC	4.00E + 4^h^	9^c,d,h^	2.54E + 2	1.02E + 7	1.5 %	4/69 (5.8 %)

The estimations of the density and diameter of neuronal classes in the cerebellar cortex are based on some literatures, as indicated in superscript letters. Total surface per cell and total surface overall assumes simple spherical cells. Chance overall indicates the unbiased random chance of encountering a piece of membrane of that particular cell type. Fraction patched indicates the number of patched neurons per cell class. MLIs and UBCs indicate molecular layer interneurons and unipolar brush cells, respectively. Uncommon cell types, such as Lugaro cells and Candelabrum cells, are excluded because of their relative rarity

^a^Ref. [21]

^b^Ref. [46]

^c^Ref. [30]

^d^Ref. [47]

^e^Ref. [48]

^f^Ref. [41]

^g^Ref. [35]

^h^Ref. [49]
